# Integrating Drought Stress Signaling and Smart Breeding for Climate-Resilient Crops: Regulatory Mechanisms and Genetic Strategies

**DOI:** 10.3390/plants14243714

**Published:** 2025-12-05

**Authors:** Mingyu Wang, Yuwei Zhao, Yaqian Huang, Jun Liu

**Affiliations:** Key Laboratory of Biology and Genetic Improvement of Oil Crops, Oil Crops Research Institute of the Chinese Academy of Agricultural Sciences, Ministry of Agriculture and Rural Affairs, Wuhan 430062, China

**Keywords:** drought stress, hormonal signaling, smart breeding, multi-omics integration

## Abstract

The escalating frequency and severity of drought events pose significant threats to agricultural productivity and food security. Drought stress not only restricts crop growth and yields but also destabilizes agricultural ecosystems. Over evolutionary timescales, plants have developed intricate adaptive strategies, encompassing drought escape (accelerated phenology), avoidance (water-conserving morphology) and tolerance (cellular protection), which involve complex biological mechanisms spanning molecular signaling, metabolic reprogramming and organ morphological remodeling. To mitigate drought risks, breeding drought-tolerant and water-efficient crops is imperative. Currently, drought resistance breeding is undergoing a paradigm shift, transitioning from traditional phenotypic selection toward genomics-assisted selection, molecular design and artificial intelligence (AI)-driven predictive modeling. This review provides a comprehensive analysis of drought stress response mechanisms in crops, integrating three key dimensions: physiological/biochemical adaptations, hormonal signaling networks and morphological/structural modifications. Furthermore, it critically evaluates recent advances in genetic improvement approaches for drought resistance, such as marker-assisted selection, transgenic technology and gene editing. It also explores the integration of multi-omics data and AI to enhance precision molecular breeding and overcome the inherent trade-off between drought resistance and yield potential. By synthesizing advancements in molecular breeding and smart agriculture, this work provides a roadmap for developing climate-resilient crops optimized through synergistic trait engineering and intelligent environmental sensing.

## 1. Introduction

With the intensification of global climate change, the frequency and severity of droughts have increased steadily, making drought one of the primarily abiotic stresses threatening agricultural production and food security [1,2]. According to the Food and Agriculture Organization (FAO) and the World Meteorological Organization (WMO), crop losses caused by extreme drought events over the past five decades have endangered the food security of hundreds of millions of people, with direct economic losses exceeding [3,4]. Drought is among the most severe natural disasters affecting China, resulting in annual grain production losses of over 10 million tons and economic losses exceeding RMB 27 billion [5]. With the progression of global warming, the area affected by drought in China has expanded, the frequency of drought events has increased and the severity of these disasters has intensified [6]. Recent studies in China have made significant contributions to this research field. For examples, knocking out *TaGW2* in wheat synergistically alters drought tolerance and seed size [7], and gene editing of *ZmVPP1* improves drought tolerance in maize [8]. Projections indicate that by mid-21st century, the global average temperatures may rise by 1.5–2.0 °C above pre-industrial levels, and precipitation patterns will become increasingly erratic [9]. These trends indicate that droughts will become more frequent, widespread, and prolonged, presenting escalating challenges to intensive, water-dependent agricultural systems.

Agricultural production currently faces dual constraints: severe climate fluctuations and growing pressures from population expansion, limited arable land, and scarce water resources [10]. The United Nations Population Fund (UNFPA) estimates that the global population could reach 9.7 billion by 2050, requiring a more than 50% increase in global food compared to current levels [11]. However, available freshwater resources are unlikely to keep pace and may even decrease in many regions [12]. In arid and semi-arid regions, particularly in Asia, Africa, and Australia, competition for water among agricultural, urban, and industrial sectors is intensifying [13].

Under drought stress, numerous physiological processes in crops across all developmental stages are suppressed, making the protection of crop productivity a central priority in global food security strategies [14]. Over time, plants in both natural and agricultural environments have evolved multilevel drought adaptation strategies, commonly categorized as drought avoidance (water-conserving traits), tolerance (cellular protection) and escape (accelerated development) [15]. Drought resistance is a complex, genetically controlled trait regulated at morphological, physiological, biochemical and molecular levels [16]. Fundamentally, these strategies entail optimizing key plant processes—including water use efficiency, stomatal conductance, osmotic adjustment, reactive oxygen species (ROS) homeostasis, and photosystem stability—to minimize the adverse effects of drought [17].

Despite significant advances in elucidating plant drought mechanisms, several key challenges remain in systematically enhancing crop drought tolerance [18,19]. First, research has disproportionately focused on model plants (e.g., *Arabidopsis* and rice), with limited studies on staple crops such as maize, wheat, and soybean—especially under field conditions. Second, many drought-related genes validated in model plants have not been sufficiently investigated for their conservation and regulatory mechanisms across species, restricting their application in major crops. Third, as a polygenic quantitative trait, drought resistance is genetically complex, rendering traditional breeding inefficient and often constrained by an unfavorable trade-off between drought tolerance and yield potential. Consequently, the breeding of high-yield, drought-tolerant crop varieties remains suboptimal.

To address these challenges, integrating advanced technologies has emerged as a crucial strategy for the sustainable improvement of crop drought adaptation. Utilizing multi-omics data (genomics, transcriptomics, proteomics and metabolomics) alongside high-throughput phenotyping and environmental big data enables precise identification of genes and regulatory elements associated with key drought-related traits [20]. Advanced gene-editing tools (e.g., CRISPR/Cas) facilitates targeted modification of critical genes, reducing reliance on foreign genes, shortening breeding cycles and enhancing environmental adaptability [21]. The integration of molecular mechanism analysis, gene editing and AI-driven predictive modeling accelerates the intelligent design and prediction of drought-resistance traits, thereby expediting the breeding process.

In summary, escalating climate change and increasing resource constraints present agriculture with unprecedented challenges. Drought stress remains the dominant limiting factor for improving and stabilizing crop yields. A comprehensive analysis of the multilevel drought adaptation mechanisms in crops will deepen our understanding of plant stress biology and drive the transition of molecular breeding toward greater precision and intelligence. While previous reviews have covered omics approaches [19,22], this work uniquely integrates AI-based predictive frameworks with practical breeding strategies, providing a roadmap for climate-resilient crop design through synergistic trait engineering. Unlike previous reviews that often focus on isolated aspects, we offer a holistic framework that emphasizes the synergy between multi-omics data, AI-driven predictive modeling and gene editing to overcome the drought-yield trade-off. Our work provides a unique systems-level integration by synthesizing drought response mechanisms across multiple scales—from molecular signaling to organ morphology—and explicitly linking them to emerging smart breeding technologies like AI and CRISPR.

## 2. Mechanisms of Crop Responses to Drought Stress

### 2.1. Drought Signal Transduction

**Figure 1:**
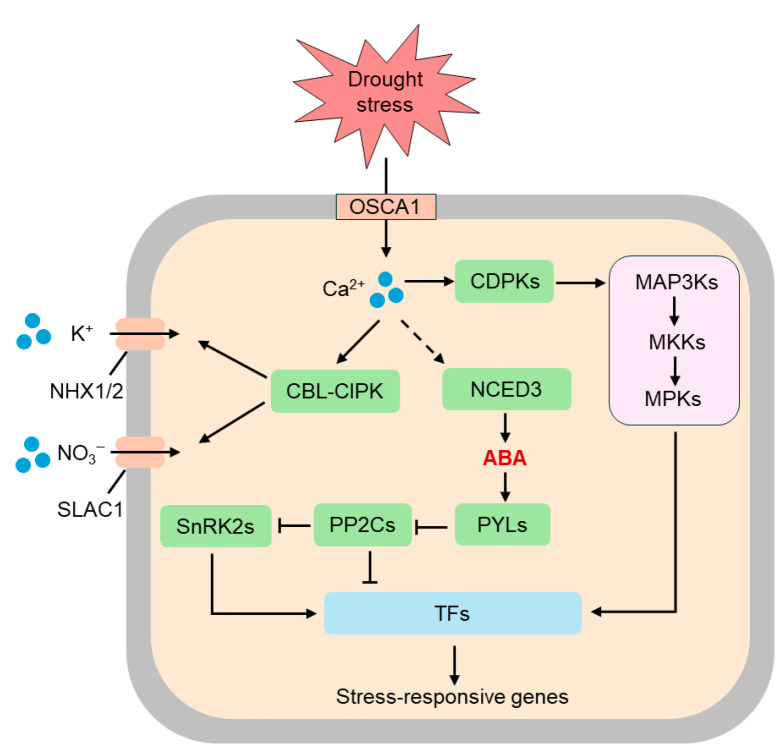
Sensing and signaling mechanisms of drought stress in plants. Plants perceive drought stress primarily through alterations in intracellular osmotic potential and mechanical strain at the plasma membrane. The osmosensor OSCA1 mediates osmotic stress-induced changes in intracellular calcium (Ca^2+^) concentration. Elevated Ca^2+^ serve as a secondary messenger, activating multiple downstream signaling pathways. Specifically, calcium signals induce the formation of the calcineurin B-like protein-CBL-interacting protein kinase (CBL-CIPK) complex, which regulates ion channels such as NHX1/2 (Na^+^/H^+^ antiporters) and slow anion channel 1 (SLAC1), thereby modulating potassium (K^+^) and nitrate NO_3_^−^ transport. In parallel, calcium-dependent protein kinases (CDPKs) and mitogen-activated protein kinase (MAPK) cascades—including MAP3Ks, MKKs, and MPKs—are activated and participate in further signal transduction. The activation of 9-cis-epoxycarotenoid dioxygenase (NCED3) promotes abscisic acid (ABA) biosynthesis, which then binds to pyrabactin resistance 1-like (PYL) receptors and modulates the activity of protein phosphatase 2Cs (PP2Cs) and SNF1-related protein kinases 2 (SnRK2s), leading to the regulation of various transcription factors (TFs). Ultimately, these integrated signaling events activate the expression of stress-responsive genes, enabling plants to adapt to drought conditions.

Crop responses to drought stress involve intricate, multi-layered pathways (Figure 1) [23]. Before physiological and morphological adaptation can occur, plants first detect and transmit signals of deteriorating water conditions via cellular molecular sensing systems. Once these signals enter the cell, they trigger a series of regulatory events—including transcriptional control, protein modification and RNA-mediated adjustment—forming a highly sophisticated molecular network. This network is supported by four principal pillars: signal perception, gene transcriptional regulation, post-translational protein modifications and non-coding RNA regulation.

#### 2.1.1. Drought Signal Perception and Primary Transduction

Plants sense drought primarily through changes in intracellular osmotic potential and mechanical strain at the plasma membrane. Mechanosensitive ion channels, such as MscS-Like (MSL) and Mid1-complementing activity (MCA) proteins, respond to membrane tension changes caused by cellular dehydration, facilitating Ca^2+^ influx [24]. Receptor-like kinases (RLKs) detect mechanical signals at the cell wall-membrane interface, initiating phosphorylation cascades [25]. Osmosensory proteins (e.g., OSCA family members) open to allow Ca^2+^ influx when extracellular water potential decreases, triggering calcium signaling waves [26]. In rice, Ca^2+^ signal transduction depends on *OsOSCA1.1*, and rice lines overexpressing *OsOSCA1.1* demonstrate enhanced resistance to hyperosmotic stress [27]. Drought also alters cellular sugar and ion concentrations, and metabolic sensors (e.g., hexokinase, HXK) activate downstream responses. Ca^2+^ signals are decoded by calcium-dependent protein kinases (CDPKs) and calmodulin-dependent kinases (CaMKs), which subsequently activate MAPK cascade signaling pathways [28]. Secondary messengers—including hydrogen peroxide (H_2_O_2_) and nitric oxide (NO)—further amplify these signals and integrate with hormone pathways (e.g., abscisic acid, ABA), forming a complex, multi-pathway signaling network [29].

#### 2.1.2. Transcriptional Regulatory Networks Involved in Drought Responses

After signal perception and transduction, information is conveyed to the nucleus, where various transcription factors (TFs) regulate the expression of drought-responsive genes. ABA-dependent transcription factors—such as the bZIP-type ABF/AREB family—bind to ABA-responsive elements (ABREs) in target gene promoters, activating the expression of osmoprotective proteins, late embryogenesis abundant (LEA) proteins and antioxidant enzymes [30]. MYB/MYC family TFs, which function downstream of ABA, regulate the synthesis of secondary metabolites (e.g., flavonoids) to enhance ROS scavenging [31]. ABA-independent transcription factors—including DREB/CBF (AP2/ERF family)—bind to dehydration-responsive elements (DREs) or C-repeat (CRT) elements, activating genes involved in both drought and cold resistance. In C_4_ crops like sorghum and maize, DREB2A is particularly critical for pre-ABA activation of defense genes [32]. The NAC family TFs control cell wall remodeling and delays leaf senescence, with a particular role in promoting deeper root growth. TFs often act in complexes (e.g., NAC × bZIP or DREB × WRKY), enabling dual regulatory pathways and multifactor synergy. Chromatin accessibility—regulated by histone acetyltransferases (HAT) and deacetylases (HDAC)—finely tunes the availability of drought-response gene clusters, a process increasingly recognized as critical for long-term drought tolerance [33].

#### 2.1.3. Post-Translational Modifications of Proteins Involved in Drought Responses

Plant drought response involves not only transcriptional regulation but also rapid and dynamic control at the protein level. SNF1-related protein kinases 2 (SnRK2) phosphorylate guard cell ion channels (e.g., SLAC1), enabling rapid stomatal closure [34]. PP2C protein phosphatases negatively regulate ABA signaling through dephosphorylation [35]. E3 ubiquitin ligases (e.g., RGLG and COP1) selectively degrade positive or negative regulatory proteins to promptly terminate signaling [36]. The 26S proteasome system rapidly degrades certain ABA pathway repressors (e.g., ABI5-binding proteins) [37]. SUMOylation enhances the stability of specific drought-responsive TFs (e.g., DREB2A) [38]. Acetylation—often combined with histone modifications—is typically associated with increased transcriptional activity, effectively upregulating stress-responsive genes [39]. Changes in subcellular localization also play a role: some phosphorylated TFs translocate from the cytoplasm to the nucleus to exert their regulatory functions [40].

#### 2.1.4. Regulation by Non-Coding RNAs Involved in Drought Responses

Recent studies have shown that non-coding RNAs not only directly regulate mRNA expression but also fine-tune key nodes in drought response signaling pathways. For example, miR398 is downregulated under drought stress, relieving its repression of superoxide dismutase (SOD) genes and enhancing ROS scavenging [41]. miR169 regulates nuclear factor Y subunit A (NF-YA) TFs, altering root growth patterns [42]. The miR156/miR172 module adjusts flowering time, enabling reproductive escape during drought [43]. Long non-coding RNAs (lncRNAs) can function as competing endogenous RNAs (ceRNAs), binding to miRNAs to indirectly increase the expression of drought-responsive mRNAs. Additionally, some lncRNAs interact with chromatin remodeling complexes to regulate the accessibility of drought-resistance gene loci.

### 2.2. Physiological and Biochemical Responses

**Figure 2:**
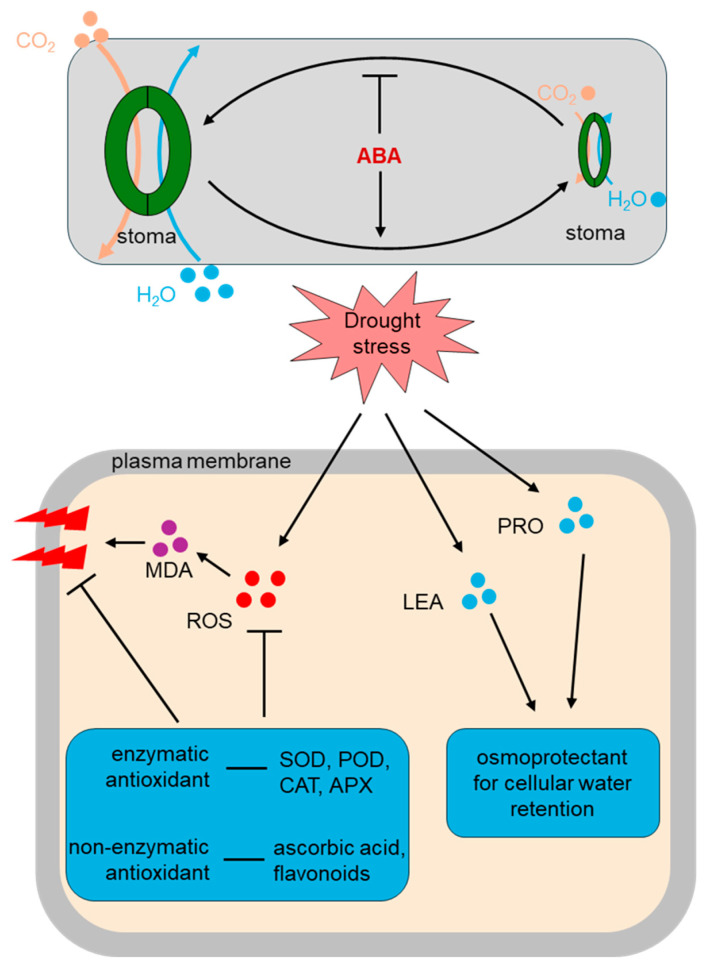
Physiological and biochemical pathways in plant drought stress response. Under drought stress conditions, plant stomata close to reduce water loss and limit CO_2_ uptake. ABA serves as a key signaling molecule that promotes stomatal closure and facilitates water conservation. Drought stress also triggers the accumulation of reactive oxygen species (ROS) within cells, resulting in lipid peroxidation and elevated levels of malondialdehyde (MDA), causing cellular membrane damage. To mitigate ROS-induced injury, plants activate antioxidant defense systems, including enzymatic antioxidants (e.g., superoxide dismutase, catalase and ascorbate peroxidase) that directly scavenge ROS, and non-enzymatic antioxidants (e.g., ascorbic acid, glutathione and flavonoids) that assist in neutralizing ROS. Additionally, the accumulation of proline and late embryogenesis abundant (LEA) proteins serves as osmoprotectants, aiding in water retention and maintenance of cell structure. Collectively, these osmoprotectant agents enhance cellular tolerance to drought stress.

Physiological and biochemical responses are central to plant drought resistance. Drought stress triggers a series of changes in plants—from the cellular to the organ level—that represent both adaptive responses to water deficit and activation of defense mechanisms. Key processes include ROS homeostasis regulation, osmotic adjustment, photosynthetic system remodeling, and water use efficiency (WUE) optimization (Figure 2).

#### 2.2.1. ROS Accumulation and Antioxidant Defense Systems

ROS exhibit dual roles: their accumulation is induced by drought, and at low concentrations, they act as signaling molecules to initiate stress responses; at high concentrations, however, they cause oxidative damage to lipids, proteins and DNA [44]. Plants maintain cellular redox homeostasis and scavenge excess ROS through the coordinated action of enzymatic antioxidant systems and non-enzymatic antioxidants [45].

Drought stress induces stomatal closure, limiting CO_2_ uptake and disrupting the photosynthetic electron transport chain. This increases electron leakage in chloroplasts and mitochondria, ultimately leading to excessive ROS production. Major reactive oxygen species (ROS) include singlet oxygen (^1^O_2_), superoxide anion (O_2_•^−^), hydrogen peroxide (H_2_O_2_), and hydroxyl radicals (•OH) [46,47].

To counteract ROS toxicity, plants have evolved efficient enzymatic and non-enzymatic antioxidant systems. The enzymatic system includes superoxide dismutase (SOD), peroxidase (POD), catalase (CAT), glutathione peroxidase (GPX), glutathione reductase (GR), and ascorbate peroxidase (APX), which work together to convert ROS into harmless molecules (e.g., H_2_O and O_2_) and maintain cellular redox equilibrium. Non-enzymatic antioxidants—including ascorbic acid, glutathione, tocopherols, carotenoids, flavonoids and phenolic compounds—directly scavenge free radicals or interrupt free radical chain reactions, indirectly protecting membrane structure and metabolic stability [48].

Studies have shown that drought-tolerant crop varieties rapidly activate ROS scavenging systems at the early stages of drought, maintaining high antioxidant enzyme activities and non-enzymatic antioxidant levels [49]. This effectively reduces membrane lipid peroxidation and malondialdehyde (MDA) accumulation. In recent years, TFs involved in antioxidant regulation (e.g., ZAT family, HSF and WRKY) have been shown to upregulate antioxidant gene expression during drought, making them important targets for drought resistance genetic improvement [50,51,52].

#### 2.2.2. Osmotic Adjustment and Cellular Water Stability

Osmotic adjustment (OA) is a key biochemical mechanism enabling plants to cope with drought. It is achieved by regulating cellular osmotic potential, promoting water uptake and maintaining cell turgor to prevent plasmolysis [53]. The core of OA lies in the synthesis and accumulation of compatible solutes such as proline, betaine, soluble sugars (e.g., sucrose, trehalose, raffinose) and polyols [54]. These compounds are low in toxicity and chemically inert, allowing them to accumulate at high concentrations without disrupting normal cellular function. Proline serves not only as a classic osmoprotectant for cellular water retention but also as a ROS scavenger and membrane protector. Under drought conditions, the expression of proline biosynthetic enzymes (P5CS and P5CR) is significantly upregulated, while the degradation enzyme ProDH is downregulated, leading to substantial proline accumulation [55]. Betaine and other quaternary ammonium compounds stabilize proteins and membranes, enhancing dehydration tolerance. In the meantime, the accumulation of osmoprotectants such as proline and betaine effectively reduces cellular osmotic potential to maintain cell turgor, preventing plasmolysis and metabolic collapse under severe dehydration [56]. Additionally, certain LEA proteins and dehydration-protective proteins help maintain cytoplasmic vitrification during extreme dehydration, stabilizing intracellular macromolecules [57].

#### 2.2.3. Remodeling Photosynthetic Systems and Regulation of Energy Distribution

Drought stress directly impacts the stability of the photosynthetic system and energy metabolism, with photosystem II (PSII) being particularly sensitive [58]. Stomatal closure limits CO_2_ availability, further impairing the photosynthetic electron transport chain. Excess excitation energy in chloroplasts cannot be used for carbon assimilation, increasing ROS production and oxidative damage to the D1 protein in the PSII reaction center [59]. To alleviate such damage, plants activate photoprotective mechanisms: (1) enhanced non-photochemical quenching (NPQ), which dissipates excess energy as heat via the xanthophyll cycle; (2) accelerated PSII repair, which increases D1 protein turnover to maintain reaction center activity; and (3) alternative electron pathways (e.g., water-water cycle and photorespiration), which transfer surplus electrons to O_2_, reducing oxidative pressure on photosynthetic tissues [60,61,62,63]. Drought also induces reduced leaf stomatal conductance to limit water loss via transpiration, while aquaporins (PIPs, TIPs) regulate intercellular water flow to optimize water distribution among tissues. This dynamic trade-off between photosynthetic CO_2_ assimilation and inhibited transpiration forms the basis for improved WUE [62].

#### 2.2.4. Precise Regulation of Water Use Efficiency (WUE)

WUE reflects the amount of assimilated carbon produced per unit of water consumed. Under drought, high-WUE varieties typically display lower stomatal conductance, moderate photosynthetic rates and a high root-to-shoot ratio—maximizing carbon assimilation per unit water [64]. This trait is shaped by stomatal control, leaf anatomy, leaf angle and biochemical factors (e.g., Rubisco activity and ribulose bisphosphate [RuBP] regeneration). At the molecular level, genes regulating stomatal development and movement (e.g., EPF/EPFL family, SLAC1, and OST1) are closely associated with WUE. The expression of aquaporin gene families (PIPs, TIPs, NIPs) also significantly influences root water uptake and mesophyll water transport. Modulating the expression of these genes can optimize water allocation and enhance photosynthetic carbon assimilation under drought, providing critical genetic resources for breeding drought-tolerant crops [65].

In summary, crop physiological and biochemical responses to drought stress are multi-layered, dynamic. Coordinated processes involve ROS regulation, osmotic adjustment, photosynthetic system stabilization and WUE enhancement. Deep analysis of these mechanisms not only can enrich our understanding of plant drought resistance biology but also establish potential targets for future genetic improvement and molecular breeding of drought-tolerant crops.

### 2.3. Organ Morphological Remodeling

At the organ level, morphological plasticity is a fundamental basis for plant drought tolerance [66]. The root system undergoes significant adaptive changes—including deeper primary roots, altered distribution of lateral roots and increased root-to-shoot ratio—to enhance access to deep soil water (a critical “resource acquisition” strategy) [67]. Leaves minimize water loss through precise stomatal regulation; some plants also reduce leaf area, adjust leaf angle or thicken the waxy cuticle in the early stages of drought to lower light absorption and evaporation, achieving water conservation. Anatomical changes in stems and vascular tissues (adjusted vessel characteristics) further optimize vertical water transport, enhancing overall drought adaptability [68,69,70].

Under drought stress, plants not only rely on physiological and biochemical regulation to maintain cellular function, but also adaptive morphological and structural changes to improve water uptake/retention, reduce transpiration, and ensure carbon assimilation and reproductive development. These changes include root architectural adjustments, leaf structural optimization, modifications of stem water storage and conducting tissues, and altered reproductive strategies—reflecting the structural mechanisms of drought resistance evolved over long periods.

#### 2.3.1. Adaptive Reconstruction of the Root System

As the primary organ for water and nutrient absorption, the root system exhibits remarkable plasticity under drought. In maize and sorghum, roots grow deeper and denser, prioritizing primary and deep lateral roots to increase the root-to-shoot ratio and explore deeper soil moisture [71]. By contrast, rice relies on adventitious root proliferation and aerenchyma formation to cope with intermittent drought [63]. Roots grow deeper and denser, with plants prioritizing the development of primary and deep lateral roots to increase the root-to-shoot ratio and explore deeper soil moisture [65]. For example, sorghum and pearl millet exhibit enhanced lateral root elongation under drought, reaching depths of 1.5–2.0 m. Drought also stimulates root hair proliferation (increasing both density and length), significantly expanding the water-absorbing surface [72]. Aquaporins regulate intercellular water movement, boosting instantaneous water uptake. Anatomical modifications—such as increased suberization and lignification of cortical cell walls—reduce radial water loss and enhance conduction. The formation of larger xylem vessels improves water transport to aboveground parts. These changes are co-regulated by ABA and auxin (IAA): ABA promotes deep rooting, while local IAA distribution modulates root apical meristem activity to guide root architecture [11,73].

#### 2.3.2. Optimization of Leaf Morphology and Anatomy

As the main site of photosynthesis and transpiration, leaf morphological adjustment is critical for drought resistance [74]. In wheat and barley, plants reduce exposure and transpiration by decreasing leaf area and narrowing leaf shape [53,75,76]. Plants reduce exposure and transpiration by decreasing leaf area and narrowing leaf shape. Leaf curling/folding—observed in crops such as maize and wheat—minimize exposed surface, reducing radiation and transpiration rates [77]. Adjusting leaf angle to a more vertical orientation alleviates midday radiation, reducing corresponding heat and transpiration stress [78]. Thicker cuticles limit water vapor loss, and changes in stomatal density—typically reduced to minimize transpiration but sometimes increased for rapid response—further refine water management. Drought may induce the formation of multiple palisade layers or thicker cell walls to improve light use efficiency and mechanical support; succulent plants increase the proportion of water-storage cells [79]. In maize, drought induces the formation of multiple palisade layers to improve light use efficiency [80].

#### 2.3.3. Structural Adaptation of Stems and Conducting Tissues

Stems play key roles in mechanical support, water storage, and transport. In CAM plants (e.g., cacti and agave), the development of water-storage tissues—composed of parenchyma cells rich in mucilage or polysaccharide—provides a critical water source during severe drought [81,82]. Drought-tolerant species often exhibit optimized xylem vessels (narrower and denser conduits) to reduce embolism risk and ensure continuous water transport under low water potential [83]. Suberization of the epidermis and cortex further limits direct water loss and evaporation from the stem surface. ABA are key regulators in vessel differentiation and secondary wall formation: ABA tends to reduce vessel diameter to mitigate embolism, while ethylene accelerates lignification [84]. In maize and sorghum, ABA tends to reduce vessel diameter to mitigate embolism, while ethylene accelerates lignification [77].

#### 2.3.4. Adjustment of Reproductive Organs and Reproductive Escape Strategy

Drought significantly impacts flowering, pollination and grain filling, prompting plants to adopt adaptive reproductive strategies. Reproductive escape is common in annuals such as chickpea and wheat, which flower 10–15 days earlier to complete their life cycle before severe drought occurs [79,80,81]. Reproductive escape is common in annuals (e.g., chickpeas), which flower and set seed early to complete their life cycle before severe drought occurs [11,85,86]. Plants optimize resource allocation by reducing the size and investment in floral structure. They may adjust the pistil- to-stamens ratio, decreasing stamen number and vitality to lower reproductive costs and prioritize female gamete development [87]. Additionally, enhanced seed dormancy and thickened seed coats improve offspring survival under adverse conditions [88].

Morphological and structural adaptations—including deeper root water uptake, reduced transpiration and water conservation in leaves and stems, and strategic adjustments in reproduction—are central to drought resistance, which form interactive networks with physiological, biochemical and hormonal regulation. These strategies are finely tuned by genetic and signaling pathways, enabling plants to survive and reproduce in harsh drought environments. Accordingly, breeding for drought tolerance requires integrating structural trait improvement and molecular regulation to achieve multi-trait system optimization.

### 2.4. Plant Hormones

Plant hormones play a central role in integrating and coordinating drought responses. ABA is the core of drought signaling and adaptation, earning it the title of “drought warning hormone” [89]. Under drought conditions, ABA rapidly accumulates and binds to pyrabactin resistance 1-like/regulatory components of ABA receptors (PYR/PYL/RCAR) receptors, mediating signal transduction to regulate stomatal closure and the expression of stress response genes (e.g., LEA proteins and antioxidant enzymes) [88]. Other hormones—including jasmonic acid (JA), ethylene (ETH) and salicylic acid (SA)—interact with ABA through complex synergistic or antagonistic relationships, collectively shaping plant drought adaptation strategies [90].

#### 2.4.1. ABA-Dependent Signaling Pathways

Under drought, roots and mesophyll cells sense water deficit via osmotic changes, mechanical signals and Ca^2+^ influx, triggering ABA synthesis [90]. Upregulation of the key rate-limiting enzyme gene *NCED* (9-cis-epoxycarotenoid dioxygenase) leads to rapid ABA accumulation in leaves and phloem, followed by its transport throughout the plant [29]. ABA binds to PYR/PYL/RCAR receptors, forming a ternary complex that inhibits PP2C phosphatase activity and activates the SnRK2 kinase family [91]. The activated SnRK2 phosphorylates downstream TFs (e.g., bZIP-type ABF/AREB), promoting the expression of stress-responsive genes (e.g., *RD29B*, *LEA* gene families, *RAB* gene families) [30,92]. This enables core physiological functions such as stomatal closure and reduced transpiration. Additionally, upregulation of osmotic adjustment enzymes enhances water retention, while activation of antioxidant systems scavenges excess ROS and stabilizes cellular metabolism. ABA signaling also directly regulates plasma membrane ion channels (e.g., SLAC1, KAT1), modulating guard cell turgor for rapid stomatal movement [93].

#### 2.4.2. ABA-Independent Pathways

While ABA is central to drought signaling, some drought response mechanisms operate independently of ABA. A prominent example is the DREB/CBF pathway: DREB TFs directly bind to DRE/CRT promoter elements, regulating genes involved in osmotic adjustment, membrane stabilization and antioxidation [94]. This pathway is typically triggered by membrane receptors and intracellular Ca^2+^ signals, amplified through MAPK cascades, and ultimately activates the transcription of drought-resistance genes [29]. In drought-tolerant crops (e.g., sorghum, millet and other C_4_ species), ABA-independent pathways are particularly critical. TFs including DREB2A, NAC, ERF and WRKY directly induce the expression of LEA proteins, dehydrins and protective enzymes, enabling plants to initiate defense responses before ABA accumulation [95,96].

#### 2.4.3. Multi-Hormone Interaction Networks

Drought adaptation is governed by complex networks of hormonal interactions. For instance, jasmonic acid (JA) cooperate with ABA via the MYC2 TF pathway to promote stomatal closure and enhance antioxidative responses. However, at certain developmental stages, JA may compete with ABA for resources, suppressing growth to prioritize survival [97]. ETH works with ABA to stimulate root elongation during mild drought; under severe drought, high ETH levels accelerate leaf senescence and jointly induce stomatal closure. SA stabilizes ROS signaling during drought to prevent oxidative damage, but excessive SA may antagonize ABA-mediated stomata control, requiring a fine balance. Levels of auxin (IAA), cytokinin (CK) and gibberellin (GA) typically decrease under drought, suppressing cell division and elongation to conserve resources for stress adaptation. Reduced GA limits unnecessary elongation and metabolic expenditure [98]. These networks enable plants to flexibly adjust their metabolic and growth strategies based on drought severity, duration and developmental stage, facilitating a shift from growth prioritization to survival.

#### 2.4.4. Downstream Response Modules and Signal Amplification

Once drought signals are transmitted to the transcriptional level, multiple downstream modules are activated: (1) the osmoprotection module, which regulates the biosynthesis of proline and betaine [99]; (2) the membrane and protein protection module, which boosts the expression of stress proteins (e.g., LEA, dehydrin, heat shock proteins [HSP]); (3) the redox homeostasis module, which activates enzymes (e.g., SOD, CAT, APX, and GPX) and non-enzymatic antioxidants [29]; and (4) the stomatal and root regulation module, which controls genes (e.g., EPF/EPFL, SLAC1, aquaporins) to balance leaf transpiration and root water uptake. Signal amplification relies on secondary messenger systems, such as Ca^2+^ oscillations, cGMP/cAMP signaling, phosphoinositide pathways, and H_2_O_2_-mediated positive feedback loops, ensuring rapid and coordinated responses.

## 3. Crop Improvement for Drought Tolerance

Developing water-saving and drought-tolerant crops is a critical strategy for mitigating drought stress [100]. With advances in breeding technologies, crop improvement has evolved from conventional hybridization to molecular breeding approaches, and now enters the “Breeding 4.0” era—integrating biotechnology, AI and big data analytics [101]. Despite these technological innovations, the central goal of breeding remains unchanged: designing and selecting crop varieties that best meet human needs. Current conventional molecular breeding methods (e.g., QTL mapping and genome-wide association studies [GWAS]) have identified numerous key genes and functional loci associated with drought tolerance [102]. However, drought stress is a complex quantitative trait regulated by multiple genes, with intricate regulatory network spanning genetic, transcriptional, protein and metabolic layers. This complexity makes it challenging for traditional molecular approaches to fully decipher and integrate these systemic responses.

### 3.1. Applications of Multi-Omics in Drought-Tolerant Germplasm Development

Multi-omics technologies—integrating genomics, transcriptomics, proteomics and metabolomics datasets—provide unprecedented power to systematically unravel the molecular basis of plant drought tolerance and accelerate the breeding process [103]. The core value of multi-omics lies in its ability to reveal the intrinsic connections between genes, metabolites and phenotypes from a holistic perspective, overcoming the limitations of traditional “phenotyping-by-guesswork” and enabling precise “design breeding.” Single-omics approaches capture only one facet of the drought response: genomics identifies potential gene variants; transcriptomics reveals expression changes; proteomics characterizes protein abundance and modifications; and metabolomics detects alterations in key metabolites involved in osmotic regulation [104,105,106,107]. By integrating multi-omics data, researchers can construct comprehensive drought regulatory networks and pinpoint critical TFs (e.g., DREB and NAC), signal proteins and effector molecules—ideal targets for gene editing and marker-assisted selection.

While GWAS and QTL mapping link drought traits to genomic regions, these intervals are often broad and contain multiple candidate genes [102]. Multi-omics data serve as efficient filters to narrow the candidate pool. For example, within a drought-associated QTL region, prioritizing genes with significant expression changes (transcriptomics), protein abundance shifts (proteomics) and key metabolite accumulation (metabolomics) greatly improves the accuracy of functional gene identification. For example, in rice, quantitative trait locus (QTL) mapping has identified *DEEPER ROOTING 1* (*DRO1*) on chromosome 9, which is involved in auxin-mediated regulation of the root system and consequently enhances rice yield under drought conditions [108]. In genome-wide association studies (GWAS) on drought tolerance in maize, natural variations in the promoter region of *ZmVPP1*, which encodes a vacuolar-type H(+)-pyrophosphatase, have been shown to affect its expression level. *ZmVPP1* confers drought tolerance to maize seedlings by enhancing both photosynthetic efficiency and root development, overexpressing *ZmVPP1* significantly increased the survival rate of maize under drought from 20% to 80% [8]. This integrated strategy—combining genomic mapping with multi-omics validation—substantially accelerates the precise identification of functional genes.

In summary, multi-omics technologies have transformed drought breeding from an opaque, unpredictable process to a transparent, analyzable and design-driven system, providing strong scientific and technological support for developing high-efficient, water-saving, drought-tolerant crop varieties.

#### Challenges in Multi-Omics Data Integration

In the process of integrating multi-omics data, data standardization and cross-platform normalization are crucial steps for achieving high-quality analysis. Omic data often exhibit high heterogeneity due to differences in sequencing platforms, sample preparation, and experimental methods, posing significant challenges to data fusion. For example, batch effects and technical biases in metabolomic data require normalization and correction through tools such as MetaboAnalyst [109]. Furthermore, differences in feature scales and data distributions across omics often limit the broad applicability of traditional bioinformatics analysis methods [110]. Currently, the use of weighted gene co-expression network analysis (WGCNA) and visualization platforms such as Cytoscape helps identify cross-omics association patterns, but there are still certain limitations in the face of issues such as missing data and noise [111]. Therefore, continuously optimizing data preprocessing procedures and developing flexible integration frameworks are important directions for enhancing the reliability of multi-omics analysis.

### 3.2. Application of Gene Editing in Breeding

Multi-omics research has uncovered a wide array of drought-associated key genes and regulatory pathways. Gene-editing technologies, especially CRISPR/Cas system, offer powerful tools for precisely modifying these targets, enabling efficient translation of basic research discoveries into breeding materials [112]. Unlike traditional breeding (relying on hybridization, backcrossing and random recombination), gene editing enables direct and targeted design of desired genotypes, making it a defining feature of the Breeding 4.0 era [113].

Gene editing has three main applications in drought tolerance breeding. First, loss-of-function editing involves knocking out negative regulators of drought tolerance to relieve suppression of drought response pathways. For example, certain ABA receptor inhibitors or suppressor proteins in the JA signaling pathway reduce drought tolerance. Using CRISPR/Cas9 to eliminate these “brake” genes allows plants to activate protective mechanisms more rapidly under drought, enhancing resilience [114,115]. Second, gain-of-function editing enhances the activity of positive regulatory factors. Unlike traditional overexpression methods (which may cause gene silencing or uncontrolled expression), gene editing enables precise regulation. For instance, promoter editing of key drought-responsive genes (e.g., *DREB/CBF* or ERF TFs) optimize their expression patterns—ensuring strong activation only under drought stress and avoiding constitutive overexpression that impairs growth. This strategy mimics the regulatory advantages of superior natural alleles, setting a model for custom-designed plant ideotypes [116,117,118]. Third, multiplex editing simultaneously modifies multiple genes in drought regulatory networks to achieve synergistic improvements in drought tolerance [118]. In tomato, multiplex genome editing of three fruit color-related genes (*PSY1*, *MYB12* and *SGR1*) mediated by CRISPR/Cas9 was used to generate tomato lines with various fruit colors from red-fruited varieties [119]. In wheat, the coordinated modification of *tagw2* and *taarr12* improved both stress resistance and seed size. Silencing the *taarr12* gene in *tagw2* knockout wheat lines resulted in larger, heavier, and more drought-tolerant seeds [7]. For example, editing genes involved in ABA signaling and ROS scavenging simultaneously can enhance both stomatal control and oxidative stress resistance.

**Figure 3:**
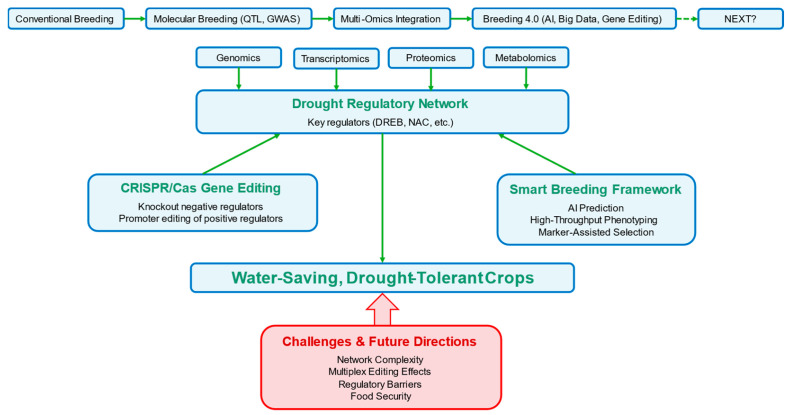
Advanced breeding strategies for drought-tolerant crop improvement. The evolutionary trajectory of crop breeding for drought tolerance spans conventional breeding, molecular breeding and multi-omics integration, culminating in next-generation approaches that incorporate artificial intelligence (AI), big data and gene editing. Multi-omics technologies (genomics, transcriptomics, proteomics, metabolomics) collectively unravel drought regulatory networks and identify key regulators (e.g., DREB, NAC transcription factors). CRISPR/Cas-based gene editing enables the targeted modification—including knockout of negative drought regulators and promoter editing of positive regulators—to enhance drought tolerance without compromising growth. The “smart breeding framework” integrates AI-driven prediction, high-throughput phenotyping and marker-assisted selection to accelerate the identification and development of drought-tolerant crop varieties. The ultimate goal is to generate water-efficient, drought-tolerant crops, while major challenges (e.g., the complexity of regulatory networks, unintended effects of multiplex gene editing, global policy barriers) and future directions (e.g., resolving drought-yield trade-offs) are also highlighted.

Despite its promise, gene editing in drought breeding faces several challenges: (1) a deeper systems-level understanding of drought response networks is needed for accurate editing target selection; (2) multiplex editing may cause unintended effects (e.g., yield penalties), requiring extensive screening and field validation; and (3) the lack of unified global regulatory policies for gene-edited crops hinders their commercialization (Figure 3).

In addition, gene editing faces several significant challenges, including off-target risks, variability in editing efficiency and regulatory approval. Off-target effects are typically caused by high sequence homology at target sites, which can lead to unintended alterations in other genomic regions, resulting in changes in gene expression, disruption of cellular processes and potentially deleterious mutations [120]. Currently, the main Cas enzymes include Cas9, Cpf1,and Cas12a, while the novel Cas12b demonstrates superior DNA targeting capabilities and a lower probability of off-target effects [120]. Selecting unique target sequences helps reduce the frequency of off-target events, but experimental constraints often pose significant challenges. In complex genomes, such as wheat, which is an allohexaploid (AABBDD), the high number of homologous genes makes it challenging to simultaneously knock out multiple homologs, resulting in lower gene-editing efficiency compared to crops like rice [120]. Various techniques have been developed for gene editing in wheat, such as engineered epegRNA to modify SpCas9 activity and nuclear localization signals [121]. At present, global acceptance of gene-edited crops is gradually increasing. In the United States and Japan, gene-edited crops are exempt from the regulations applied to genetically modified organisms (GMOs). The first commercialized gene-edited crop, the Sicilian Rouge High GABA tomato, was developed in Japan. Similarly, in 2024, Europe passed legislation to relax restrictions on gene-edited crops. India differentiates between gene-edited and genetically modified crops. China has issued the “Guidelines for the Evaluation of Gene-Edited Plants for Agricultural Use,” providing a more detailed regulatory framework for the approval of gene-edited crops.

In summary, gene-editing technology transforms the vast genetic insights obtained from multi-omics research into actionable “design blueprints” for breeding, greatly accelerating the development of efficient, water-saving, drought-tolerant “smart crops”. The combined application of gene editing, marker-assisted selection, genomic prediction and high-throughput phenomics forms the core technical framework for future intelligent breeding, offering promising solutions to global food security challenges posed by climate change.

## 4. AI-Driven Predictive Frameworks for Smart Breeding Under Drought Stress

With the development of high-throughput sequencing technology and phenomics, the amount of data generated from crop drought-resistant breeding has grown exponentially, exhibiting characteristics of high dimensionality, multimodality, and heterogeneity. Traditional statistical analysis methods, such as linear regression, often struggle to handle these complex nonlinear relationships. Artificial intelligence, especially machine learning (ML) and deep learning (DL), provides a new paradigm for analyzing complex drought-resistant mechanisms and predicting crop performance under drought stress [122,123]. AI-driven prediction frameworks are transforming breeding from an “experience-dependent” to a “data-driven” approach, with the core being to mine potential biological patterns from massive data through algorithms, thereby assisting breeding decisions.

### 4.1. AI-Enhanced High-Throughput Phenotyping and Stress Identification

Phenotype precision identification is a bottleneck in drought-resistant breeding. The integration of AI and computer vision (CV) technology has made it possible to identify and quantify drought stress symptoms at an early stage. Deep learning models based on convolutional neural networks (CNNs) have been widely applied to analyze RGB, multispectral, hyperspectral, and thermal imaging data [124,125].

In drought resistance assessment, AI models can not only identify visible morphological changes (such as leaf curling and wilting) but also capture physiological signals that are imperceptible to the human eye. For instance, by utilizing thermal infrared imaging combined with machine learning algorithms (such as support vector machine (SVM) or random forest), it is possible to accurately monitor changes in canopy temperature, thereby inversely estimating stomatal conductance (Gs) and transpiration rate, enabling real-time prediction of crop water status [126]. Furthermore, AI algorithms based on 3D point cloud data can reconstruct root system architecture (RSA), automatically extracting key drought resistance traits such as root length, root surface area, and branching angle, overcoming the challenge of “invisibility” in traditional root research [127]. These AI-driven phenotypic analyses not only enhance screening throughput but also significantly reduce phenotypic errors, providing high-quality standardized data for genetic analysis.

### 4.2. Deep Learning for Genomic Selection (GS) in Drought Tolerance

Genomic Selection (GS) is one of the core technologies in modern breeding. Traditional GS models, such as GBLUP, are typically based on linear assumptions, making it difficult to capture complex nonlinear interactions between genes (epistasis) and between genes and the environment (G × E). However, these interactions play a decisive role in drought resistance, a quantitative trait.

Deep learning algorithms, such as deep neural networks (DNNs) and recurrent neural networks (RNNs), have demonstrated superiority in genomic prediction of drought resistance traits due to their powerful nonlinear modeling capabilities [128]. Studies have shown that when dealing with large-scale genomic data containing millions of SNP markers, deep learning (DL) models can automatically learn the hierarchical structure of features and effectively capture non-additive genetic effects. For example, the Multi-task Learning framework is used to simultaneously predict yield, biomass, and water use efficiency (WUE) under drought stress, leveraging the correlation between traits to improve prediction accuracy [129]. By integrating environmental covariates (such as temperature, rainfall, soil moisture) into the input layer, AI models can construct predictive networks for genotype-environment interactions, thereby predicting the performance of specific genotypes in the target drought environment and achieving “environment-specific” variety design.

### 4.3. Integrating Multi-Omics Data via Machine Learning

Single-omics data often only reflects one aspect of drought resistance mechanisms. The core advantage of AI technology lies in its powerful ability to integrate multi-source data. By constructing a multimodal machine learning framework, researchers can integrate genomics, transcriptomics, metabolomics, and proteomics data into a unified predictive model [130].

**Figure 4:**
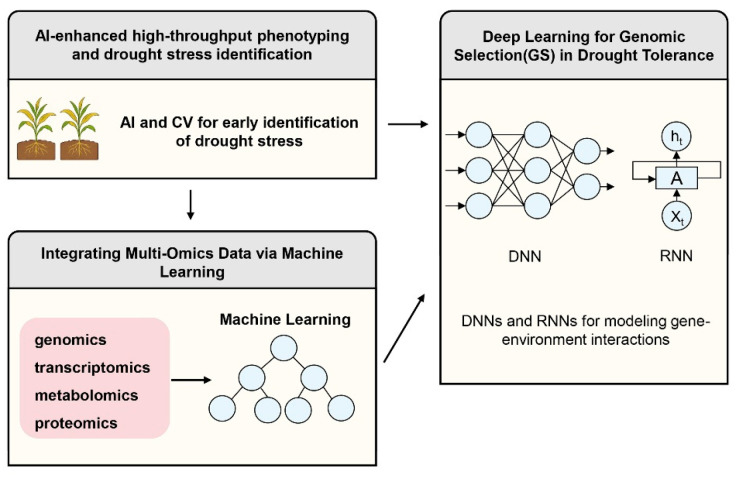
AI-Driven Predictive Frameworks for Drought-Resistant Breeding. AI-driven predictive frameworks are emerging as pivotal tools in breeding for drought resistance. These systems facilitate AI-enhanced high-throughput phenotyping by leveraging Computer Vision (CV) for the early detection and quantification of drought stress. In the context of Genomic Selection (GS) for drought tolerance, deep learning architectures, specifically Deep Neural Networks (DNNs) and Recurrent Neural Networks (RNNs), are utilized to model complex gene-environment interactions (G × E). Furthermore, the integration of multi-omics data—spanning genomics, transcriptomics, metabolomics, and proteomics—is achieved through advanced machine learning algorithms; notably, Graph Neural Networks (GNNs) are employed to conduct robust biological network analysis, thereby elucidating systemic stress responses.

For example, using Random Forest or Gradient Boosting Decision Tree (GBDT) algorithms to rank feature importance can identify biomarkers most relevant to drought resistance from thousands of genes and metabolites. This method not only identifies key regulatory genes (such as transcription factors) but also reveals systematic changes in metabolic pathways. Furthermore, biological network analysis based on Graph Neural Networks (GNNs) can model genes, proteins, and metabolites as nodes and their interactions as edges, thereby predicting the dynamic response of the drought signaling network at the systems biology level [131,132]. This whole-system prediction model can simulate phenotypic outcomes after gene knockout or overexpression, guiding the selection of gene-editing targets, thus avoiding the high cost of blind trial and error in traditional reverse genetics (Figure 4).

In summary, AI is not merely a data analysis tool, but also a predictive framework that permeates the entire process of drought-resistant breeding. It bridges the gap between microscopic molecular mechanisms and macroscopic field performance, offering intelligent solutions to address the trade-off between drought resistance and yield.

## 5. Conclusion and Future Perspectives

This review systematically synthesizes the sophisticated, multi-layered mechanisms by which crops perceive and respond to drought stress, encompassing signal perception and transduction, physiological and biochemical regulation, morphological and structural adaptation and intricate hormone network crosstalk. Drought tolerance is revealed to be a complex, polygenic trait that necessitates a systems biology approach, integrating genomics, transcriptomics, proteomics and metabolomics to accurately identify key genes and regulatory hubs.

Gene-editing technologies (e.g., CRISPR/Cas) provide robust tools for targeted improvement of drought-related traits, enabling the modification of promoter regions, knockout of negative regulator and enhancement of positive regulator. However, a persistent challenge in breeding drought-tolerant crops lies in balancing drought tolerance with yield potential. Recent field trials demonstrate concrete outcomes: under drought conditions, the survival rate of genetically edited wheat varieties decreased from 90.3% to 32.7%, but the seed size increased by approximately 5% [7]. Knocking out *OsRAV11*/*12* in rice can increase the survival rate of rice under drought from 57% to 85% [133]. Knocking out *RRS1* in rice crop increases water use efficiency by approximately 16% and promotes root development, thereby enhancing drought tolerance [134]. Future research should focus on three critical areas: (1) further elucidating the molecular basis of the drought-yield trade-off to inform breeding strategies that optimize both traits; (2) promoting the integration of multi-omics and AI-driven breeding approaches to enhance the precision and efficiency of trait selection; and (3) achieving synergistic improvements in drought resilience, high yield and environmental adaptability through innovative breeding techniques. These efforts will not only support agricultural sustainability but also ensure global food security in the face of escalating climate change and resource constraints.

## Data Availability

No new data were created or analyzed in this study.
